# Supplementary material for: A Comprehensive Review of the Contribution of Mitochondrial DNA Mutations and Dysfunction in Polycystic Ovary Syndrome, Supported by Secondary Database Analysis
Source: Int J Mol Sci. 2025 Jan 29;26(3):1172. doi: 10.3390/ijms26031172 (PMC11818232; doi:10.3390/ijms26031172)
Supplement: Supplementary file 1 [file ijms-26-01172-s001.zip › ijms-3429620-supplementary.pdf]

Supplementary Material: Search Strategy and Inclusion Criteria

This review undertakes a comprehensive narrative synthesis of the existing literature concerning mtDNA copy number, mtDNA mutations, and their associated repair and replication mechanisms in the context of PCOS. A systematic search was conducted for articles published up to September 30, 2024 using electronic databases, such as PubMed (https://pubmed.ncbi.nlm.nih.gov/) and Google Scholar (https://scholar.google.com/). The search strategy employed a combination of the following keywords: "polycystic ovary syndrome," "mitochondria," "mitochondrial DNA mutation," "mitochondrial DNA copy number," "mitochondrial DNA damage," "replication errors," "oxidative stress," "transition," and "transversion." Boolean operators, as detailed in Supplementary Table S2, were utilized to refine and optimize the search query. Eligible studies were limited to original research articles published in English, as well as pertinent literature referenced in review articles. Exclusion criteria included duplicate publications, studies unrelated to the topic, and those published in languages other than English. During the initial screening phase, records were identified through electronic database searches, and duplicates were removed. Irrelevant studies were excluded based on a preliminary review of titles and abstracts. In the final selection phase, full-text articles were meticulously evaluated to exclude those lacking comprehensive data or studies without mtDNA mutation or repair data or pathophysiological relevance. The selected studies were independently reviewed by the authors through a rigorous full-text assessment process. Any discrepancies or uncertainties in study selection were resolved through discussion and consensus. A detailed flowchart depicting the study selection methodology, including inclusion and exclusion criteria, is presented in Supplementary Figure S1.

Supplementary Table S2. The keyword and search term combinations

Search mode	The keyword and search term combinations
Search term 1	polycystic ovary syndrome OR PCOS
Search term 2	mitochondrial function OR mitochondrial DNA
Search term 3	damage OR mutation OR copy number
Search term 4	oxidative stress OR reactive oxygen species OR ROS
Search term 5	replication errors OR repair
Search term 6	transition OR transversion
Search	Search term 1 AND Search term 2
	Search term 1 AND Search term 2 AND Search term 3
	Search term 1 AND Search term 4
	Search term 1 AND Search term 2 AND Search term 4
	Search term 2 AND Search term 3 AND (Search term 5 OR Search term 6)
	Search term 1 AND Search term 2 AND Search term 3 AND (Search term 5 OR Search term 6)

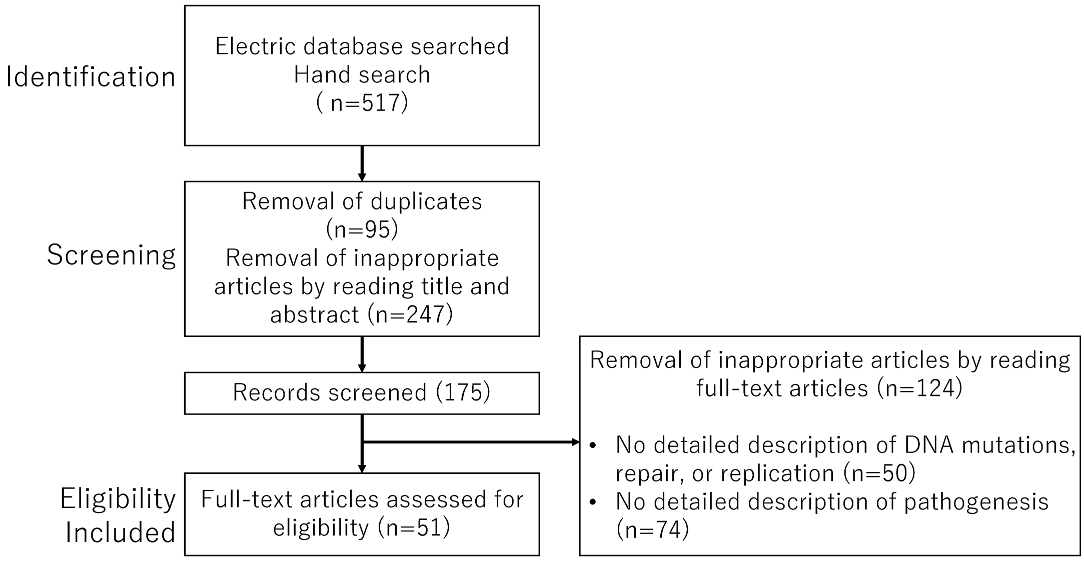

Supplementary Figure S1. The total number of articles retrieved through the search using specific keyword combinations.
